# The RNA Splicing Response to DNA Damage

**DOI:** 10.3390/biom5042935

**Published:** 2015-10-29

**Authors:** Lulzim Shkreta, Benoit Chabot

**Affiliations:** Département de Microbiologie et d’Infectiologie, Faculté de Médecine et des Sciences de la Santé, Université de Sherbrooke, Sherbrooke, QC J1E 4K8, Canada; E-Mail: lulzim.shkreta@usherbrooke.ca

**Keywords:** DNA damage response, R-loops, RNA binding proteins, pre-mRNA, splicing factors, alternative splicing, splice site selection, chromatin, transcription, signal transduction

## Abstract

The number of factors known to participate in the DNA damage response (DDR) has expanded considerably in recent years to include splicing and alternative splicing factors. While the binding of splicing proteins and ribonucleoprotein complexes to nascent transcripts prevents genomic instability by deterring the formation of RNA/DNA duplexes, splicing factors are also recruited to, or removed from, sites of DNA damage. The first steps of the DDR promote the post-translational modification of splicing factors to affect their localization and activity, while more downstream DDR events alter their expression. Although descriptions of molecular mechanisms remain limited, an emerging trend is that DNA damage disrupts the coupling of constitutive and alternative splicing with the transcription of genes involved in DNA repair, cell-cycle control and apoptosis. A better understanding of how changes in splice site selection are integrated into the DDR may provide new avenues to combat cancer and delay aging.

## 1. Introduction

**Figure 1:**
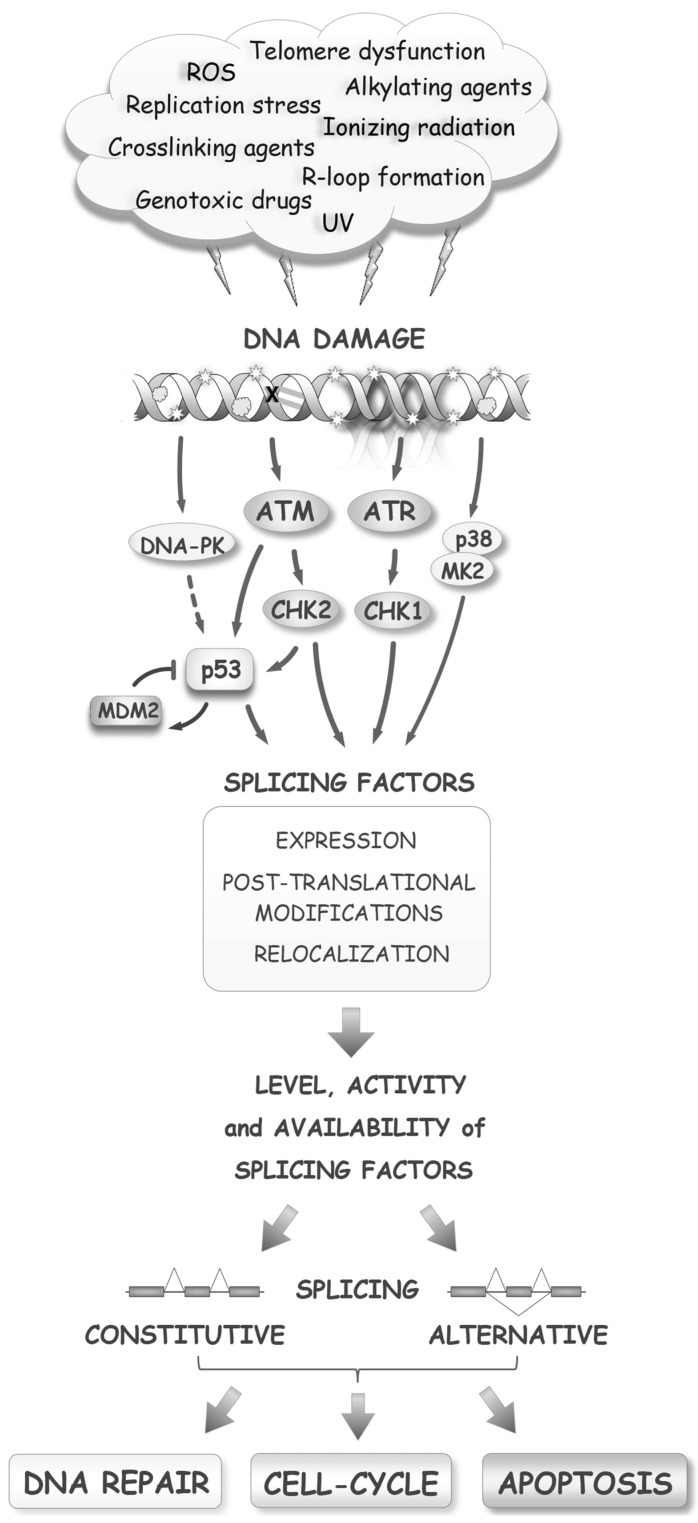
The RNA splicing response to DNA damage. Several early and late steps of the DNA damage response alter processes that impact the activity of splicing factors, ultimately affecting the production, through splicing, of components that maintain genome integrity and control cell fate.

Genomes are continuously bruised by products of normal cell metabolism and by errors in DNA replication. The frequency of DNA lesions increases when cells are exposed to UV light, gamma rays or toxic chemicals (Figure 1). While DNA damage impacts nearly every aspect of gene expression, including transcription and translation, more attention has recently been devoted to studying how DNA damage affects post-transcriptional events, with several excellent reviews published on this topic [1,2,3,4,5]. Here we review the interconnections that exist between DNA damage and pre-mRNA splicing. Following a brief overview of the basic principles of the DNA damage response (DDR), splicing and alternative splicing control, we outline how DNA damage affects the post-translational modification, localization, expression and activity of splicing factors. We then present examples showing that DNA damage often disrupts splicing by interfering with its coupling to transcription. Finally, we summarize a growing body of data that document the impact of DNA damage on the splicing and alternative splicing of genes intimately associated with the DDR and with cell fate.

### 1.1. The DNA Damage Response

DNA damage is a cause of cancer, and its accumulation is associated with organismal aging. Paradoxically, provoking DNA damage by irradiation, DNA intercalating drugs, crosslinking agents or topoisomerase inhibitors is a common strategy to treat patients suffering from cancer. This anti-cancer approach is based on the expectations that (1) an excessive number of lesions will overwhelm the DNA repair machinery of cancer cells and trigger apoptosis; and (2) that the normal and cancer cells that acquire a lesser load of mutations will not become cancerous or evolve toward more aggressive behavior, respectively.

Distinct repair mechanisms are used to correct different types of DNA lesions. Mismatch repair and base-excision repair act on simple lesions, while nucleotide excision repair, non-homologous end-joining and homologous recombination (HR) deal with more complex lesions. DNA lesions, if not adequately repaired, impede transcription and replication and result in mutations or chromosomal rearrangements. To deal with DNA lesions, the cell must mount a response that is based first on sensing the DNA lesions themselves or their direct consequences, such as blocks in replication and transcription. DNA damage triggers the phosphorylation of the histone variant H2AX, the modifications of canonical histones, and the recruitment of poly(ADP-ribose) enzymes. Depending on the type of lesions, different axes of the DDR are activated. For example, double-strand breaks (DSBs) recruit the ATM and DNA-PK kinases, while single-stranded breaks (SSBs) recruit the ATR kinase. Following their activation, the ATM/ATR kinases activate other kinases, including CHK1 and CHK2 [6] (Figure 1). These early events ramify into a signal transduction cascade that mobilizes downstream pathways to mediate cell-cycle arrest, DNA repair, and apoptosis, if the intensity of the damage is excessive. Damage sensing and many steps of the DDR rely on a variety of post-translational modifications (e.g., phosphorylation, poly(ADP-ribosylation), acetylation, methylation, ubiquitylation, sumoylation) to guide the interactions between DDR factors and components of the repair and cell-cycle machinery [7]. In addition to these initial stages, the DDR initiates a slower route aimed at modulating transcription, of which the main contributor in the ATM/CHK2 axis is p53. The p53 protein regulates expression of cyclin-dependent inhibitor protein 1A (CDKN1A or p21), apoptotic proteins (e.g., BAX and PUMA) and DNA repair components. Importantly, the E3 ubiquitin ligase MDM2 represses p53 as part of a cyclic strategy that senses the progress of damage repair [8]. When p53 activity is defective, as is often the case in cancer cells, the cell-cycle checkpoint response to damage is rewired through the p38/MK2 pathway [9]. Thus, DNA damage elicits a complex signaling cascade that coordinates cell-cycle progression and DNA repair, and reconfigures gene expression at multiple levels.

While the expression of genes that encode sensing factors, and components of the DNA repair, cell-cycle and apoptotic machineries is controlled by the p53 activation branch of the DDR, these genes also produce splice variants that harbor potentially distinct, and sometimes, opposite activities. For example, the recruitment to chromatin of cyclin D1a, but not its splice variant cyclin D1b, is sufficient to activate the DDR [10], and *Bcl-x* produces variants with pro-survival or pro-death activities [11]. A change in splicing control elicited by the DDR therefore has the potential to provide feedback on every step of the DDR and regulate repair and cell fate.

### 1.2. Splicing and Alternative Splicing

**Figure 2:**
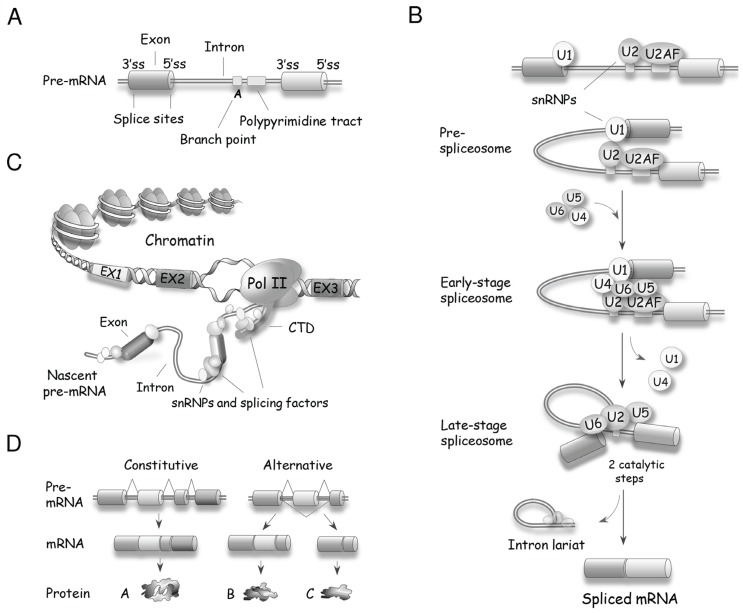
Basic principles of pre-mRNA splicing. (**A**) Schematic structure of a pre-mRNA with the position of core signal sequences that define exons and introns. ss: splice site; (**B**) A snRNP-biased view of spliceosome assembly leading to two catalytic steps that produce the mRNA and the excised intron. U2AF is a heterodimer made of the U2AF2 (U2AF65) and U2AF1 (U2AF35) proteins that respectively recognize the polypyrimidine tract and the AG dinucleotide at the 3' splice site [15]; (**C**) Spliceosome assembly is often coupled with transcription, with the carboxyl-terminal domain (CTD) of RNA polymerase II recruiting splicing components that are deposited on the nascent pre-mRNA; (**D**) In contrast to constitutive splicing, alternative splicing produces different mRNAs from a single kind of pre-mRNA.

Precursor (pre)-mRNA splicing is the process by which introns are removed from a pre-mRNA and exons are joined to produce a mature mRNA. Removal of introns from pre-mRNAs occurs in eukaryotes from yeast to human. The majority of introns in the budding yeast *Saccharomyces cerevisiae* are found in ribosomal protein genes, which produce approximately 90% of the pre-mRNAs in growing cells [12]. In mammals, except for histones and a few other genes, nearly all RNA polymerase II-transcribed genes contain introns. Splicing is performed by the spliceosome, a large nuclear macromolecular complex that contains five small nuclear ribonucleoproteins (snRNPs) (U1, U2, U4, U5 and U6) and more than 150 accessory proteins [13,14,15,16,17]. Fewer than 0.5% of human introns are processed by a minor form of spliceosome that uses the functionally homologous U11, U12, U4atac and U6atac snRNPs. The U5 snRNP is used in both spliceosome types [18]. Spliceosome assembly is a dynamic process initiated by the recognition of splice sites (Figure 2A,B); the U1 snRNP recognizes the 5' splice site, while the U2AF proteins and U2 snRNP interact with the 3' splice site and the branch site, respectively [14]. Once the borders of the intron are defined, the pre-assembled U4/U6.U5 tri-snRNP is recruited and, with the help of auxiliary proteins, the U1 and U4 snRNPs are displaced to allow U6 and U2 snRNPs to form a catalytically competent core that positions the branch point adenosine for the first of two cleavage steps. The first step produces a free upstream exon and a lariat intron covalently linked to the downstream exon. Following further spliceosome rearrangements, the second step of splicing leads to the excision of the lariat intron and the ligation of both exons. The efficiency of spliceosome assembly is increased when it is coupled to transcription [19], at least in part because the CTD of RNA polymerase II recruits spliceosome components to facilitate their deposition on the nascent pre-mRNA [20] (Figure 2C).

A single type of pre-mRNA can be spliced in different ways (*i.e.*, by inclusion of specific exons in the final spliced product) to generate distinct mRNAs (Figure 2D). This process is named alternative splicing, and is a major contributor to transcriptomic and proteomic diversity in higher eukaryotes. In humans, nearly all multi-exon primary transcripts are alternatively spliced [21,22]. On average, a human gene is made up of 8–10 exons [23], and examples of the diversifying power of alternative splicing range from two to several thousand variants from a single gene (e.g., the *Drosophila DSCAM* gene produces over 38,000 splice variants [24]). Although much remains to be done to document the remarkable diversity of functions resulting from alternative splicing, examples of functionally relevant splice variants are continuously being reported, and are found in all cellular processes [25]. The production of proteins displaying different functions is expected to be tightly controlled. Indeed, profiles of alternative splicing vary in a tissue-specific manner [26], and are often altered in diseases, including cancer [27,28,29].

**Figure 3:**
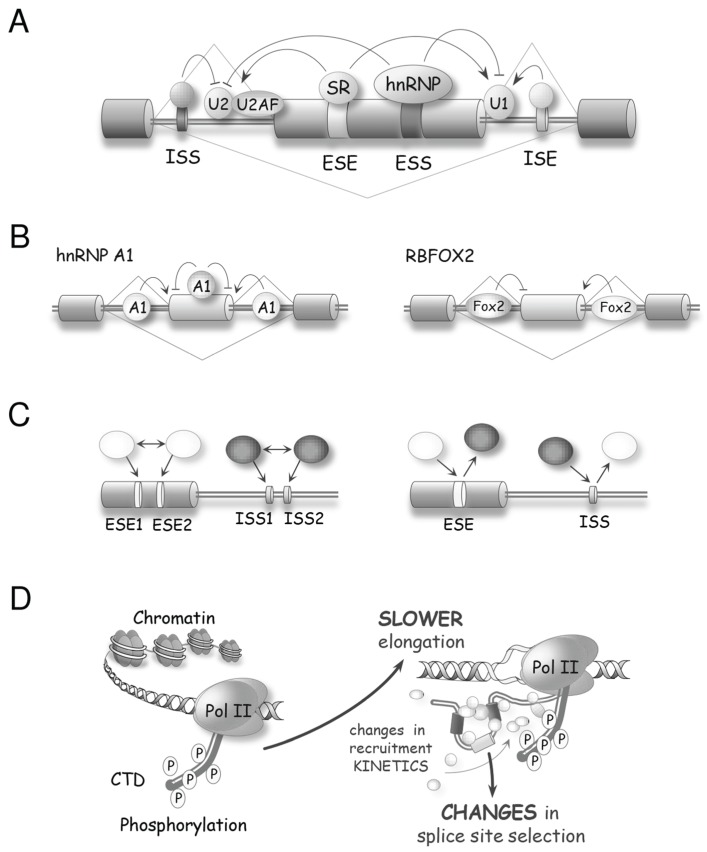

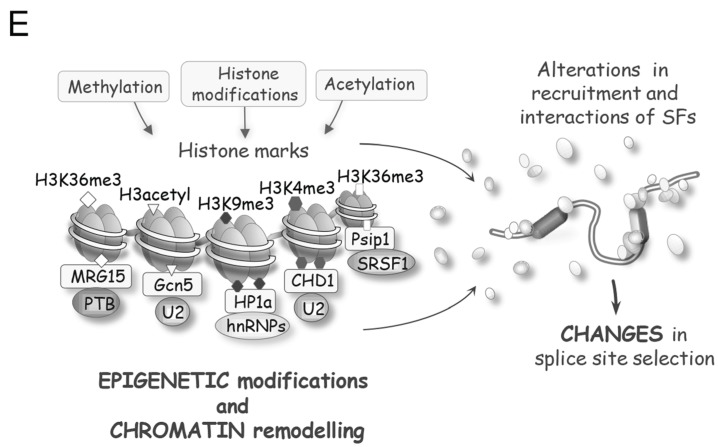
Molecular mechanisms controlling splice site selection. (**A**) A variety of splicing regulators, including hnRNP and SR proteins, bind to exon or intron splicing enhancers (ESE or ISE, respectively) and to exon or intron splicing silencers (ESS or ISS, respectively) to control splice site recognition and utilization; **(B**) The activity of splicing regulators is often position-dependent. For example, hnRNP A1 often acts as a repressor when bound to exons but can enhance splicing when bound to introns. RBFOX2 is associated with exon skipping when bound upstream of that exon, but triggers exon inclusion when bound downstream of the exon; (**C**) Depending on the identity of the factors that recognize them, the combinatorial configuration of regulatory elements leads either to synergy (left) or to antagonism (right) [30]. Inhibitory and stimulatory factors are shown as black and white, respectively; (**D**) The structure of the chromatin and the phosphorylation status of the CTD of RNA polymerase II affect the speed of transcription, which in turn impacts the time given for the binding and assembly of regulatory complexes, hence affecting splice site selection [39]; (**E**) Post-translational modifications of chromatin components and chromatin remodeling activities alter the recruitment of adaptor proteins and splicing regulators to modulate alternative splicing.

Sophisticated mechanisms that regulate alternative splicing profiles are also emerging (Figure 3). Alternative splicing units usually have weak splice sites whose utilization is controlled by sequence elements recognized by RNA binding proteins (RBPs) that act positively or negatively to recruit spliceosome components or prevent spliceosome assembly [30,31]. For example, SR proteins can interact with exonic enhancer sequences to antagonize the activity of a nearby splicing silencer element [32]. The activity of exonic silencers is often mediated by hnRNP proteins of which A1, L and PTBP1 have received most of the attention [33,34,35,36]. The respectively positive and negative functions of SR and hnRNP proteins bound to exons are often reversed when they bind to introns. For example, the binding of an SR protein near the branch site prevents U2 snRNP binding [37], while the binding of hnRNP A1 and H in introns stimulates splicing [38]. Transcript-specific studies and global analyses of alternative splicing indicate that positive and negative interactions between splicing factors with a wide range of sequence specificities play an important role in splicing regulation [30] (Figure 3A–C). In addition, since most introns are removed in a cotranscriptional manner, the control of alternative splicing is often coupled to the local state of the chromatin. Histone marks and chromatin remodeling factors impact the speed of transcription elongation and the recruitment of splicing regulators to alter splice site selection (Figure 3D,E) [39]. Thus, while great progress has been achieved in documenting the function of individual regulatory proteins, we need to better understand how these splicing factors combine their activity to control specific splicing decisions, how these activities are integrated with transcription and chromatin structure, and how they are affected by various cellular inputs and environmental insults. As is often the case in biology, insight can be provided by disrupting homeostasis. Below we will present examples of how DNA damage, by modifying the expression, localization and activity of spliceosomal proteins and splicing regulators, is providing precious information on the rules and mechanisms that control distinct steps of splice site selection.

## 2. DNA Damage Modifies Splicing Proteins

The post-translational covalent modification of DDR proteins plays a critical role in mounting the response to DNA damage [7]; it is therefore not surprising that many of the changes that affect splicing factors following DNA damage also occur at the post-translational level. Such modifications alter the steric or electrostatic profile of proteins, and provide ways to modulate their activity by modifying their interaction with other proteins or with RNA. Below, we review cases where DNA damage has been associated with the post-translational modifications of splicing factors to affect their localization, stability and activity. The emerging concept is that many of these modifications represent the initial steps of a concerted mechanism that coordinates the RNA splicing response to DNA damage.

### 2.1. PARylation

Poly(ADP-ribose) polymerases (PARPs) utilize NAD to synthesize poly(ADP-ribose) polymer (PAR) varying from 2 to 200 ADP-ribose units [40]. The activity of PARPs is counteracted by PARG, which hydrolyzes PAR. The addition of PAR, or PARylation, by the PARP1, PARP2 and PARP3 enzymes is an early event associated with DNA damage repair [41]. The PARylation of chromatin components is used to recruit DNA repair factors [6,42]. PAR is also bound by the splicing factors NONO (aka p54^nrb^), hnRNP A1 and RBMX (aka hnRNP G) [43,44], suggesting that PARylation mobilizes splicing factors at sites of damage. The binding of SRSF1 to PAR inhibits its phosphorylation [45]. Splicing factors and regulators are also substrates for PARP1 [46,47], and the PARylation of hnRNP proteins inhibits RNA binding to alter alternative splicing [48]. After treatment with methylmethane sulfonate (MMS), peroxide, ultraviolet (UV) or ionizing radiation (IR), PARylation occurs on many splicing factors (see Figure 3) [49]. While the PARylation of THRAP3 promotes its relocalization to nuclear speckles [49], it is unclear if this modification contributes to the observed exclusion of THRAP from DNA damage sites [50]. Overall, the recruitment of splicing factors by PAR and their subsequent PARylation likely represent important early events associated with the detection of DNA lesions. However, the consequence of these interactions and their impact on the splicing of DDR-related genes need to be investigated in more detail.

### 2.2. Arginine Methylation

Many splicing factors contain arginine residues that are methylated by arginine methyl transferases [51], and some factors, such as Sam68, RBM15, EWS, hnRNP A1/A2 and hnRNP K, are implicated in the DDR. Interestingly, the methylation of two arginines in hnRNP K prevents the PKCδ-mediated phosphorylation of a flanking serine, and mutating these two arginines increases the genotoxic effect of the topoisomerase II (TOP2) inhibitor etoposide, suggesting that arginine methylation of hnRNP K has anti-apoptotic function [52]. While hnRNP K normally represses the production of pro-apoptotic Bcl-xS [53], the role of arginine methylation on the splicing function of hnRNP K remains to be evaluated.

### 2.3. Acetylation

Proteins implicated in splicing are often acetylated (e.g., addition of the chemical group COCH_3_) [54], and this modification has been associated with their activity [55]. Following treatment of U2OS cells with the TOP2 inhibitor etoposide, changes in acetylation have been observed on many splicing factors (Figure 3) [50]. Likewise, cisplatin decreases the hyperacetylation of SRSF2 by TIP60 (aka KAT5) in SAOS2 cells, contributing to its stabilization [56]. The impact of cisplatin on TIP60 also favors the nuclear translocation of SR protein kinases SRPK1/SRPK2 that leads to an increase in the phosphorylation of SRSF2 [56]. BCLAF1 (a protein that interacts with BRCA1 to recruit spliceosome components following DNA damage) is rapidly deacetylated following the treatment of U2OS cells with IR [57]. Thus, the fact that acetylation can alter the activity of splicing factors warrants a broader assessment of the role of DNA damage-induced acetylation in splicing.

**Figure 4:**
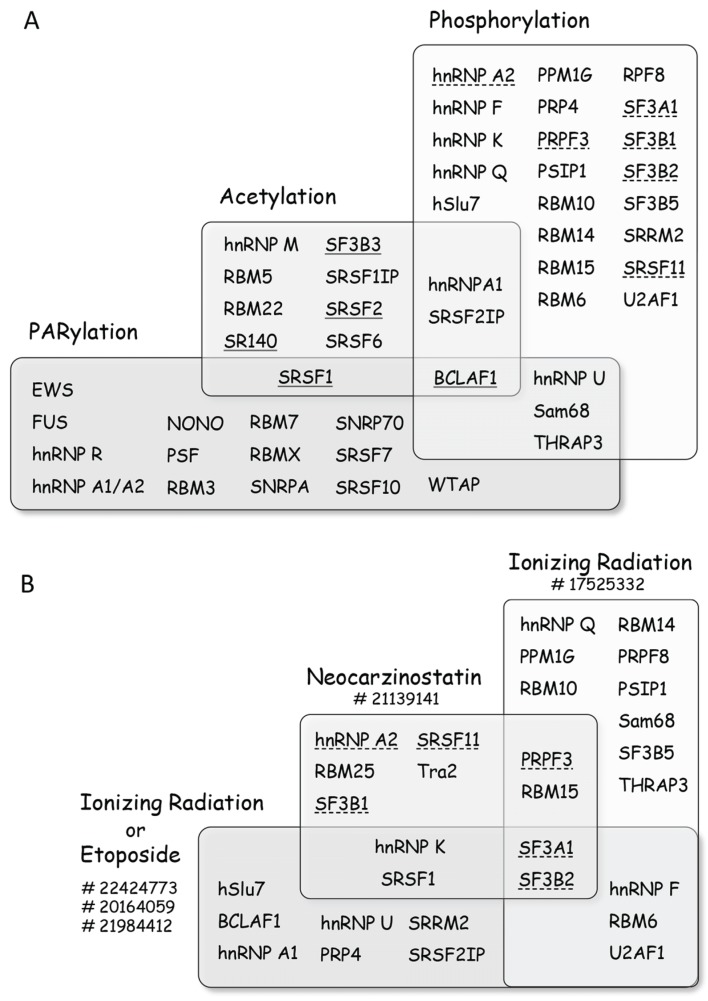
Post-translational modifications of splicing factors promoted by DNA damage. (**A**) Diagram representing splicing factors that are modified upon treatment of cells with genotoxic agents. The list is non-exhaustive and more factors can be found in the references given in the text. Splicing factors are grouped according to the type of modifications they sustain, with BCLAF1 being subjected to all three types of modifications. When DNA damage elicits deacetylation the name of the splicing factor is underlined, whereas a dashed underline indicates dephosphorylation; (**B**) Diagram representing the impact of selected treatments on the phosphorylation/dephosphorylation of splicing factors (a dashed underline indicates dephosphorylation), with SF3A1 and SF3B2 being dephosphorylated in all groups of treatments. PubMed Identification (PMID) numbers for the different studies are indicated next to treatments. The impact of other agents on specific factors is discussed in the text.

### 2.4. Ubiquitylation and Sumoylation

Sumoylation, or conjugation with a small ubiquitin-like modifier (SUMO), has been associated with DNA damage [58]. For example, treatment with the topoisomerase I (TOP1) inhibitor camptothecin delocalizes TOP1 from the nucleolus and promotes its sumoylation [59]. Many RBPs, including hnRNP proteins, are sumoylated [60]. Notably, SRSF1 is a cofactor for the SUMO E2 conjugating enzyme UBC9, and it interacts with the SUMO E3 ligase PIAS1 [61]. hnRNP K is a substrate for the ubiquitin E3 ligase MDM2 and is de-ubiquitylated upon DNA damage [62], and also sumoylated to affect its p53 transcriptional co-activator function [63]. Although SRSF1 stimulates the sumoylation of Sam68 [61], it is not known whether DNA damage affects the sumoylation of Sam68, and whether the splicing functions of Sam68 and hnRNP K are affected by sumoylation.

### 2.5. Phosphorylation

Phosphorylation is tightly associated with the DDR since the sensing of DNA lesions rapidly activates ATM, ATR and downstream kinases. A large-scale proteomics study identified more than 700 proteins that become phosphorylated at ATM/ATR consensus sites following treatment with ionizing radiation (IR) [64]. The list contains several splicing factors (Figure 4). A similar study in yeast identified PRP19 as a target for Mec1 and Tel1 (orthologs of the human ATR and ATM kinases, respectively) [65]. While PRP19 (aka Pso4) is a *bona fide* splicing factor in yeast, in mammals it is implicated in transcription elongation through the recruitment of TREX components [66]. Other proteomic studies focusing on the ATM pathway identified targets for more downstream kinases and phosphatases, or occurring at sites that are modified later during the response [50,67]. IR or etoposide affects the phosphorylation of many splicing factors (Figure 4). Another large-scale proteomic study using the radiomimetic agent neocarzinostatin (NCS) identified hundreds of phosphorylation and dephosphorylation events (40% of them independently of the ATM pathway (Figure 4) [68]. Hyperphosphorylation of SRSF1 also occurs when cells are treated with UV light or etoposide [69]. While the ATM-dependent increase in the phosphorylation of hnRNP K co-activates p53 transcription [70], it is not yet known if its function in alternative splicing is also altered. Splicing factors that are substrates of CDK1 (which is activated by ATR) include hnRNP M, MATR3, RBM7, RBM14 and SRRM2 [71].

Global proteomic studies have therefore clearly established splicing factors as targets for the DDR kinases. While there is considerable overlap in the identity of proteins that get modified by different types of agents, these studies have also revealed targets that are specific to the type of treatment or to the cell line used [50,67,71]. Global studies have rarely addressed the impact of phosphorylation events on splicing. Nevertheless, the impact of specific kinases that are activated by DNA damage on the splicing or the alternative splicing of specific genes has been examined in a few cases. Cisplatin promotes the nuclear translocation of SRPK1/SRPK2, which increases the phosphorylation of a hypoacetylated form of SRSF2 to regulate *Casp8* splicing [56]. In *Drosophila*, DNA damage by IR or camptothecin alters the alternative splicing of *Taf1* in a CHK2-dependent manner [72]. The activation of CHK2 by IR in human cells in turn activates CDK11 to stimulate the splicing of a reporter gene [73]. Oxaliplatin and cisplatin also elicit an ATM/CHK2-dependent response that alters the alternative splicing of *Bcl-x* [74]. Camptothecin reduces the level of Tra2 in *Drosophila* cells in an ATR-dependent manner [75]. CHK2 has also been linked with the phosphorylation of HuR, which affects *hTra2*β splicing and decreases the level of the hTra2β protein [76]. In human cells, Tra2 proteins favors the production of the long splice variant of CHK1, and a drop in Tra2 increases the level of phosphorylated γH2AX and reduces cell viability [77]. UV irradiation, by changing the localization of the splicing factor EWS, promotes the expression of a splice mRNA variant of *Chek2* that lacks the initiation codon, hence reducing the level of CHK2 kinase [78]. Thus, while DNA damage activates kinases that impact the alternative splicing of downstream DDR genes, splicing regulation may feedback on the signaling genes themselves, possibly as part of mechanisms to amplify the response or facilitate cell recovery.

Splicing decisions are often taken while the pre-mRNA is still being transcribed [31,39]. Post-translational modifications elicited by DNA damage occur on protein components of the transcription machinery that alter speed or pausing to impact splice site selection. For example and as discussed in more details below, exposure to UV alters the level of phosphorylation of RNA polymerase II to affect transcription elongation and splicing [79]. In addition to changes in the RNA polymerase complex, DNA damage also leads to the modification of histones and histone-binding proteins to alter nucleosome positioning and global chromatin structure [80]. These changes may in turn modulate the speed of transcription and the interaction with chromatin components of the splicing machinery to affect splicing [39,81].

## 3. The Depletion of Splicing Factors Causes DNA Damage

While irradiation and genotoxic compounds are primary causes of DNA damage, DNA lesions also occur when basic processes are deficient. A genome-wide siRNA screen demonstrated that the depletion of many splicing factors increases DNA damage, as monitored by the phosphorylation of H2AX [82]. One explanation for this result is based on the impact of depleting or mutating components of the yeast THO-TREX complex that normally coats nascent polymerase II transcripts to couple transcription and pre-mRNA maturation with mRNA export [83]. Nascent RNA sequences that are not bound by RNA binding proteins (RBPs) hybridize to the template strand of the melted DNA to form R-loops, a structure that slows transcription and promotes mutations and hyper-recombination [84,85]. Because THO-TREX components have equivalents in higher eukaryotes, with a similar impact on genomic instability when depleted [86], their depletion or that of additional splicing factors, such as SR and hnRNP proteins, may create similar problems with similar impact on genome stability [87,88]. Indeed, SR proteins are important participants in the maintenance of genomic stability. The inactivation of SRSF1 provokes the accumulation of R-loops, which lead to DNA breaks, mutations and chromosomal rearrangements that activate ATM [89,90]. The depletion of SRSF2 also promotes genomic instability [88], but the mechanism remains unclear since overexpression of SRSF2, in contrast to that of RNPS1 [91], cannot rescue defects caused by the loss of SRSF1. TOP1 also collaborates with SRSF1 to prevent R-loop formation [92]. The observations that TOP1 phosphorylates SR proteins [93] and other splicing factors, including PSF and NONO [94], and that the kinase activity of TOP1 is inhibited by the topoisomerase inhibitor camptothecin [93], suggest that camptothecin may promote genomic instability not only by blocking TOP1 function in DNA topology but also possibly by modulating the activity of splicing factors.

**Figure 5:**
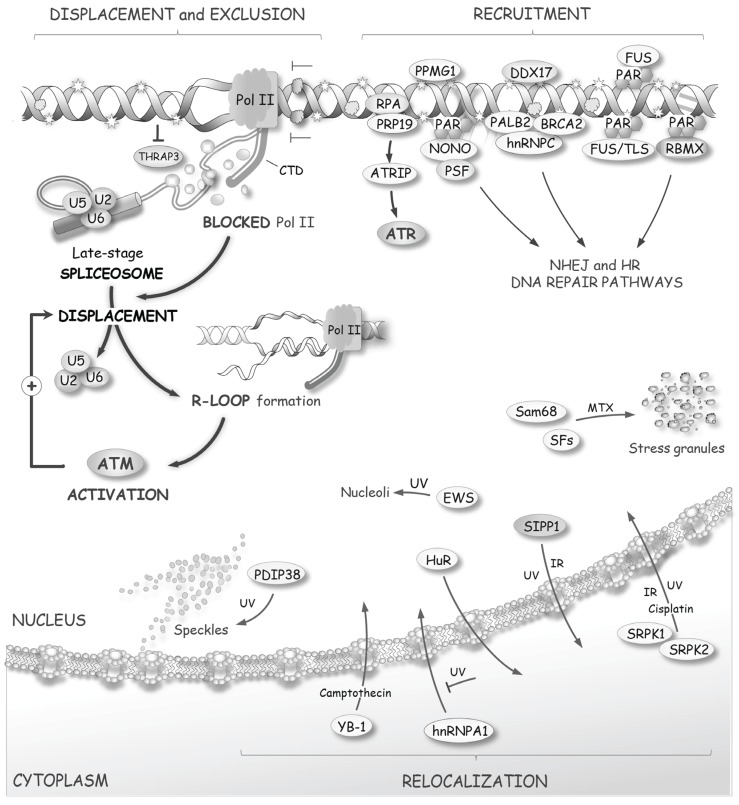
DNA damage affects the distribution of splicing factors. As described in the text, some splicing factors are recruited at sites of damage to participate in sensing or DNA repair. On the other hand, nascent pre-mRNAs that are associated with transcription complexes blocked at lesions will be stripped of late-spliceosome components to form RNA/DNA duplexes (R-loops) that activate ATM. DNA damaging agents also affect the subcellular localization of several splicing factors.

The R-loop-mediated genomic instability caused by reducing the level of THO-TREX components and SR proteins may also occur when the activity of other RBPs are affected, potentially amplifying DDR signaling and splicing changes, as when late-stage spliceosomes are displaced after RNA polymerase II encounters DNA lesions (Figure 5 and see below). Consistent with this view, both the use of the splicing inhibitor pladienolide B, which targets components of the SF3B complex, and the depletion of BUGZ and BUB3, which interact with U2AF and SF3A3, promote R-loop formation and activate the DDR [95]. The splicing regulator hnRNP A1 is implicated in telomere biogenesis [96]. Although hnRNP A1 displays affinity for telomerase RNA and single-stranded DNA telomeric repeats [97,98], it also interacts with TERRA transcripts synthesized from telomeric repeats to contribute to telomere capping and genome integrity [99]. However, whether the depletion of hnRNP A1 activates the DDR through R-loop formation and telomere dysfunction remains to be evaluated.

## 4. Exclusion and Recruitment of Splicing Factors at Sites of Damage

DNA lesions are deleterious if they allow the misincorporation of nucleotides that lead to protein malfunction. If the affected protein is involved in DNA repair directly or in cell-cycle control, this may lead to more instability and damage. Transcriptional stalling at sites of lesions may also generate replication stress. It is therefore crucial for the cell to recognize sites of damage, to limit local transcription and to provide access to the repair machinery. Given that transcription is silenced at sites of DNA damage [100], the association of splicing factors at these sites is expected to decrease. Consistent with this view, THRAP3, a factor that interacts with splicing factors to couple transcription with splicing [101,102], is excluded from DNA damage sites (Figure 5), correlating with loss of transcription and decreased mRNA processing [50]. Interestingly, DNA lesions that block transcription (elicited by UV irradiation, but not by oxidative damage, DSBs or DNA inter-strand crosslinks) rapidly decrease the localization of U2 and U5/U6 snRNPs at irradiated sites (Figure 5). Because the U1 and U4 snRNPs are not similarly displaced, this form of damage suggests the specific loss of late-stage spliceosomes [103]. Notably, spliceosome displacement elicits R-loop formation, which in turn activates ATM, further enhancing the displacement of splicing components, and providing the necessary signaling output to reconfigure splicing and alternative splicing globally [103]. The mechanism that triggers the dissociation of late-stage spliceosomes is unknown. Since it is known that the phosphatase PPM1G (aka PP2Cγ) regulates splicing positively and is recruited at DNA damage sites [50,104,105], it would be of interest to test whether PPM1G contributes to the DNA damage-induced delocalization of THRAP3 and the disengagement of late-stage spliceosomes.

While DNA damage can prevent the association of some splicing components, other observations indicate that splicing factors are recruited at sites of damage. However, it is important to indicate that sites of damage are often experimentally defined as large chromatin regions that recruit massive amounts of proteins such as γ-H2AX to form nuclear foci visible by light microscopy, and that proteins recruited to foci are unlikely to be all in the immediate vicinity of the DNA lesion itself [80]. Thus, recruitment of splicing factors does not necessarily imply a function in repair but may be part of a strategy to coordinate repair with splicing decisions.

An early step associated with the recruitment of repair proteins is the transient polymerization of PAR. Because several hnRNP proteins and some SR proteins display affinity for PAR [46,106], these proteins may be rapidly recruited at sites of DNA damage. The splicing regulator RBMX associates with DSBs in a PAR-dependent manner [107,108] (Figure 5). The siRNA-mediated depletion of RBMX sensitizes cells to DNA-damaging agents that use homologous recombination (HR) for repair (e.g., IR, camptothecin, mitomycin C, chlorambucil, oxaliplatin and carboplatin) but also to UV and *tert*-Butyl hydroperoxide (tBHP), all of which cause lesions not primarily repaired by HR [108]. The mechanism by which RBMX contributes to HR remains intriguing since the localization of RBMX at sites of damage is not required for repair [108]. However, the depletion of RBMX, as well as the depletion of several other pre-mRNA processing factors, affect BRCA2 expression [108], suggesting that RBMX may be post-translationally modified when recruited at sites of damage, and then released to affect the alternative splicing of target genes [109,110,111,112,113,114,115,116]. The screen that identified RBMX as contributing to HR also identified other RNA splicing factors involved in HR, including U2 snRNP proteins SF3A3 and SF3B1, and U5 snRNP protein PRP8 [108]. Depleting some of these proteins reduced BRCA2 expression, but the impact of these factors and of RBMX on BRCA2 splicing was not tested [108]. DDX17 also localizes at sites of DSB repair, and is known to regulate the alternative splicing of genes encoding DNA- and chromatin-binding factors [117], as well as components of the regulatory network of androgen and estrogen receptors [118,119].

The splicing regulator FUS [120] is also recruited to DSBs in a PAR-dependent manner [121] (Figure 5). Likewise, the fused oncogenic version FUS/TLS is mobilized at lesions created by oxidative DNA damage in a PARP1-dependent manner [122]. A role for FUS in DSB repair is suggested by the observation that ablation of FUS in mice confers high sensitivity to IR [123]. Familial mutations in FUS linked to amyotrophic lateral sclerosis (ALS) decrease an interaction with the histone deacetylase HDAC1 and produce a FUS protein defective in DNA repair and DDR [124]. Many ALS-causing mutations in FUS result in mislocalization of FUS to the cytoplasm in interaction with U1 snRNP [125], supporting the view that ALS cells are deficient in their ability to repair DNA. The ablation of the related protein EWS also confers high sensitivity to IR [123]. Importantly, EWS plays a critical role in regulating the changes in alternative splicing induced by UV light, and partially relocalizes to the nucleolus upon UV irradiation, an event not seen when cells are treated with NCS or etoposide [78]. The EWS-interacting factor YB-1 has also been implicated in DNA repair [126,127,128].

Other splicing factors are multifunctional proteins that play a direct role in DNA repair. For example, hnRNP C partially localizes to sites of DNA damage following IR, and this interaction occurs in association with PALB2 and BRCA2 [129] (Figure 5). hnRNP C antagonizes the activity of U2AF65 (U2AF2) to prevent portions of Alu elements to be recognized as exons [130], and its depletion has a broad impact on alternative splicing [131]. Since hnRNP C associates with the SWI/SNF chromatin remodeling complex that controls alternative splicing [132,133], hnRNP C may mediate some of its splicing effects cotranscriptionally. hnRNP C therefore contributes to the repair of DNA lesions directly through interaction with the repair proteins PALB2 and BRCA2, and more globally by regulating the splicing of genes that encode components of the HR repair machinery (BRCA1, BRCA2, RAD51 and BRIP1) [129]. RBM14, a protein structurally related to hnRNP A1, may also play a direct role in DNA repair by controlling the efficiency of NHEJ through an interaction with Ku80 that is stimulated by IR [134].

Another multifunctional protein is the splicing and ubiquitin E3 ligase protein PRP19. PRP19 is essential for DNA repair in yeast [135], and in mammals it interacts with CDC5L and the repair helicase WRN [136]. DNA damage promotes the ubiquitylation of PRP19, which then dissociates from CDC5L [137]. PRP19 recognizes DNA damage through RPA bound to ssDNA [138,139]. PRP19 then ubiquitylates RPA to recruit ATRIP and activate ATR [138,139] (Figure 5).

The pleiotropic PSF protein is mobilized to lesions after laser-induced DNA damage [140] (Figure 5). PSF displays *in vitro* DSB end-rejoining activity [141], and is implicated in NHEJ and HR repair pathways [140,142,143]. PSF forms a complex with NONO, a protein that binds to PAR and is implicated in NHEJ and HR [43,144]. PSF also associates with the nuclear matrix and splicing regulator MATR3, which itself interacts with PTBP1 [140,145]. The depletion of MATR3 increases the retention of PSF at DNA damage sites [140].

Thus, the available data suggest that the localization of splicing factors at sites of damage is affected in different ways. Some factors are expelled to block splicing, allowing formation of R-loops and amplification of the DDR signaling pathway; other splicing factors are recruited to act directly in DNA repair, while others are recruited in the vicinity of lesions to be modified, and released to execute more downstream portions of the DDR.

## 5. DNA Damage Relocalizes Splicing Factors

Although DNA damage impacts the association of splicing factors at sites of lesions, it also affects their subcellular localization (Figure 5). For example, UV irradiation triggers the cytoplasmic retention of hnRNP A1, with an impact on alternative splicing [146,147]. Notably, doses of UV light that do not trigger this A1 relocalization also have an impact on splicing, most likely by affecting the phosphorylation and elongation speed of RNA polymerase II [79]. UV irradiation and IR also redistribute the putative splicing factor SIPP1 to the cytoplasm [148].

The TOP2 inhibitor mitoxantrone (MTX) redistributes Sam68, along with other splicing regulators, to nuclear stress granules in PC-3 cells and promotes splicing changes in genes regulated by Sam68 [149]. However, this pathway is independent of the DDR signaling cascade. The siRNA-mediated depletion of Sam68 sensitizes LNCaP cells to cisplatin-induced apoptosis [150]. Cisplatin and the depletion of Sam68 in LNCaP cells shift splicing in favor of pro-apoptotic Bcl-xS [74,150,151]. It is unclear whether DNA damage affects the cellular distribution of Sam68 to reduce its recruitment at the *Bcl-x* gene locus. The activity of Sam68 in *Bcl-x* splicing requires hnRNP A1 [152,153], whose localization is also affected by genotoxic stresses [146,147].

In MRC-5 cells, PDIP38 helps install specialized polymerases at UV-damaged sites to mediate repair [154]. In HeLa and A549 cells however, UV irradiation translocates PDIP38 not to UV repair foci but to nuclear speckles where its association with splicing components may help control the alternative splicing of *Mdm2* [155].

Cisplatin provokes the nuclear accumulation of SRPK1 and SRPK2 kinases to increase the phosphorylation of SR proteins [56]. IR and reactive oxygen species (ROS) also elicit the nuclear accumulation of SRPK2 [156]. On the other hand, UV irradiation induces a dynamic redistribution of SRSF1, SRSF9, SRSF7, U1-70K, hTra2β and NONO to areas around nucleolar fibrillar components [157].

HuR has been implicated in splicing control [158], and DNA damage triggers its export to the cytoplasm through the phosphorylation of CDK1 by the CHK1 and CHK2 pathways [159,160,161]. While exposure of HCT116 cells to ROS-generating sodium arsenate stimulates the CHK2- and p38-mediated phosphorylation of HuR, in this case it stimulates the binding of HuR to the nuclear pre-mRNA encoding the splicing regulator hTra2β to alter its alternative splicing [76].

Camptothecin relocalizes YB-1 from the cytoplasm to the nucleus where it loses its ability to interact with EWS and participate in cotranscriptional splicing [2,162]. On the other hand, UV, but not etoposide, elicits the partial relocalization of EWS to the nucleolar compartment [78].

Thus, while DNA damage affects the localization of several splicing factors, the nature of the lesions and the cell line used may determine which splicing factors are affected.

## 6. DNA Damage Alters the Expression of Splicing Factors

Early studies revealed the negative impact that DNA damage has on global transcription [163,164]. However, further analyses indicated that the steady-state levels of specific transcripts or their products were increased [165], possibly reflecting mRNA stabilization and/or stimulation of translation [166]. The impact of DNA damage-induced changes in transcription, mRNA stability and translation on the expression of splicing factors is difficult to predict because splicing factors are often drafted to accomplish a variety of functions, sometimes in different cellular compartments [167]. Nevertheless, adjusting the level of spliceosome components and splicing regulators may be important after DNA damage. For example, the overexpression of RBM17 in the A2780 ovarian carcinoma cell line is associated with broad resistance to DNA damage-inducing drugs through the splicing of genes transcriptionally regulated by ESR2 [168]. Increases in the mRNA and protein levels of hnRNP proteins have been noted to occur following DNA damage [169,170,171,172,173]. Mitomycin C increases the expression of SRSF6, SRSF1 and SRSF2 in U2OS-derived cells [174]. UV also increases the level of SRSF1 in MCF-7 cells to modulate *Mdm2* splicing; however this increase in SRSF1 is not due to increased mRNA levels but by a change in the alternative splicing of *SRSF1* that affects protein abundance [175,176]. The treatment of HCT116 cells with camptothecin also affects the alternative splicing of genes encoding splicing factors including RBM8A (aka Y14), the branch site protein SF1, SF3A1, U2AF1, hnRNP A2, SIP1 and SRSF8 to produce transcripts lacking important functional domains [177]. Global proteomics studies have noted only a few changes in the level of splicing factors. For example, the treatment of U2OS cells with etoposide leads to a two-fold decrease in levels of QKI [50].

Interestingly, a recent study has revealed an intimate relationship between DNA damage at telomeres and the expression of splicing factors [178]. Persistent telomere dysfunction in mice elicits a hematopoietic defect that mimics human myelodysplastic syndromes [178]. Defective hematopoietic stem cells display ATR-dependent DNA damage signaling that reduces the expression of U2AF2, SRSF2, SRSF10, SF3B2 and SF3A3, and globally alters the alternative splicing of genes involved in maintaining genome stability, DDR, chromatin remodeling and histone modifications. The functional impact of these changes is supported by the fact that nearly one-third of them should produce non-functional proteins, mainly because of premature stop codons. Notably, downregulating the expression of SRSF2 in mice hematopoietic cells alters the alternative splicing of DNA repair and telomere maintenance genes and induces telomere dysfunction that may exacerbate splicing defects [178].

Translational control may represent a more efficient way than transcription to control the production of splicing factors, particularly when only a short-term adaptation is needed before lesions are repaired. Another strategy to control the level of splicing factors is through their alternative splicing. SR and hnRNP proteins are exquisitely designed to respond in this manner since each group contains alternative exons with premature stop codons (PTC), allowing rapid fine-tuning of protein levels through nonsense-mediated RNA decay (NMD) [179]. Consistent with this view, the alternative splicing changes induced by DNA damage often target genes encoding splicing factors with PTC-containing exons [180].

## 7. Impact of DNA Damage on Constitutive Splicing

Although DNA damage was initially associated with global drops in gene expression and RNA processing activities, both positive and negative impacts have now been documented. While it may be important to decrease transcription and splicing at sites of lesions to facilitate sensing and access by the DNA repair machinery, broader splicing alterations may be required to optimize the production of DNA repair enzymes, to implement more stable adjustments in the cell-cycle or to initiate the apoptotic program. Notably, UV irradiation reduces the splicing efficiency of individual introns in a dozen genes including *Akt1*, *Fancg*, *Atr*, *Atm*, *Aurka* and *Aurkb* in an ATM-dependent manner [103], although the functional impact of these events is unclear. On the other hand, etoposide stimulates the removal of introns in genes encoding the DNA repair components ATRIP, BACH and EXO1 [181]. The molecular pathway at work in this case involves the ATM/ATR-dependent phosphorylation of serine-1423 in chromatin-bound BRCA1 to promote its interaction with BCLAF1, itself in association with several spliceosomal proteins including U2AF, SF3B1 and PRP8, to stimulate splicing and increase the production of repair factors [182]. Consistent with the importance of this route for mounting an appropriate DDR response, phosphorylation of serine-1423 in BRCA1 confers resistance to IR and is associated with cell-cycle arrest [183]. Moreover, the siRNA-mediated depletion of BRCA1, BCLAF1 or U2AF increases sensitivity to IR and etoposide, and results in defective DNA repair and genomic instability in MCF-7 and 293T cells [181]. Thus, the noted increase in the mRNA level of DNA repair genes associated with genotoxic stresses [184] may be the consequence, at least in part, of more efficient pre-mRNA splicing.

A similar outcome was reported for two genes involved in cell-cycle control. First, resistance to DNA damage and improved splicing of *Cdkn1a* (encoding the CDK inhibitor p21) are stimulated by SKIP, a protein that interacts with SNIP1, THRAP3, BCLAF1, U2AF and PRP19, and also associates with the *Ccnd1* gene to increase the levels of cyclin D1 [101,185]. Thus, the association of BCLAF1 with spliceosome components [186] may coordinate the splicing of both DNA repair and cell-cycle genes following DNA damage. The fact that BCLAF1 is a target for several types of post-translational modifications elicited by DNA damage (Figure 4A) is consistent with this central regulatory position.

## 8. DNA Damage Alters Transcription-Coupled Splicing Decisions

As discussed above, several features of transcription affect splice site selection in different ways (Figure 3D,E) [31,39]. The elongation speed and pauses of a transcribing RNA polymerase determine how much time is given for enhancer or repressor complexes to assemble on a nascent pre-mRNA and influence the commitment of competing pairs of splice sites. This speed of elongation is altered by (1) the phosphorylation the carboxyl-terminal domain (CTD) of the large polymerase II subunit; (2) the association of elongation factors like P-TEFb; and (3) the modification of chromatin components including histones. In addition, interactions between components of the RNA polymerase complex and spliceosomal factors increase splicing efficiency and alter splice site selection. Finally, chromatin remodeling components and epigenetic changes on histones modulate the interaction with adaptors that recruit splicing regulators. Below we present DDR-relevant examples in these categories.

• UV treatment of Hep3B and HCT116 cells changes the phosphorylation of the CTD of RNA polymerase II to affect transcription elongation and alternative splicing in an ATM/ATR-independent manner [79]. On the other hand, and as discussed above, lesions created by UV block transcription leading to the dissociation of late-stage spliceosomes [103].
• SR proteins regulate splicing decisions by interacting directly with exon and intron sequence elements on pre-mRNAs [30,31,187]. More recently however, SRSF2 was implicated in the release of the transcription elongation factor P-TEFb from the repressor 7SK RNA at paused transcription sites [188]. A similar function for hnRNP A1/A2 in the release of P-TEFb and transcription elongation has been reported [189]. Since DNA damage promotes changes in the level of SRSF2 and in the localization of hnRNP A1 [56,146], splicing alterations may possibly occur through P-TEFb-mediated effects on transcription elongation.
• The RNA polymerase II-associated protein EWS confers resistance to IR and UV light [78,190], and mice lacking EWS are hypersensitive to IR [123]. These phenotypes may be due, in part, by the fact that a deficiency in EWS affects the alternative splicing of *cyclin D1* [191], *Fas* [192], *Mdm2* [162], and the DNA repair genes *Abl1*, *Chek2* and *Map4k2* [78]. EWS interacts with spliceosome components, including the U1 snRNP protein U1C [193], the branch site protein SF1 [194] and YB-1 [162,195]. UV decreases the association of EWS with target RNAs [78]. Camptothecin impairs the interaction between EWS and YB-1, possibly affecting spliceosome assembly to provoke exon skipping in *Mdm2* and other genes [162]. Although camptothecin treatment leads to hyperphosphorylation of the CTD of RNA polymerase II [162,196], the transcription elongation inhibitor DRB prevents camptothecin-mediated polymerase II phosphorylation, but not the impact of camptothecin on splicing, suggesting that a change in transcription elongation is not responsible for the observed shift in *Mdm2* splicing. While the impact of camptothecin on *Mdm2* splicing is independent of p53 [162], it is unclear how it promotes a loss of interaction between EWS and YB-1. UV irradiation partially relocalizes EWS to the nucleolus, but this effect is not seen with the TOP2 inhibitor etoposide [78].
• The epigenetic histone mark H3K36me3 is required to recruit the mismatch repair machinery and for HR-mediated repair [197,198]. H3K36me3 is elevated in nucleosomes residing on exons relative to those found in introns [199,200,201,202], and a splicing inhibitor or splice site mutations alters the deposition of the H3K36me3 mark [203]. The preferential association of H3K36me3 with coding sequences may therefore be used to prioritize the repair of coding portions of the genome. Notably, the chromatin-associated protein PSIP1 interacts with H3K36me3 and with several splicing regulators including SRSF1, SRSF2, SRSF10, hnRNP proteins and snRNP helicases [204]. Moreover, silencing the expression of the short splice variant of PSIP1 changes the localization of SRSF1 and impacts alternative splicing [204]. DNA damage leads to the phosphorylation of PSIP1 [64], but it is not known if this alters the interaction of PSIP with H3K36me3 or with splicing regulators to affect splicing. Interestingly, the splicing factor protein SF3B1, whose expression is altered by DNA damage and whose activity is linked to DNA repair, preferentially associates with nucleosomes residing on exons to modulate splicing [205]. If DNA damage promotes the dissociation of splicing components from exon-specific nucleosomes, this could represent a strategy for the unobstructed sensing of local damage and the recruitment of the repair machinery.
• Several large non-coding RNAs (lncRNAs) provide binding platforms for splicing regulators [206,207] and factors that modify chromatin to alter splice site selection [208]. DNA damage affects the transcription of the lncRNAs TUG1, Panda and lincRNA-p21 [209,210]. Panda sequesters transcription factors induced by DNA damage and prevents apoptosis when cells are treated with doxorubicin [211]. LincRNA-p21 recruits hnRNP K to control the transcription of p53-dependent genes [212], and enhances sensitivity to radiotherapy [210]. Whether the interaction of hnRNP K with lincRNA-p21 is affected by DNA damage to impact hnRNP K-mediated splicing events is not yet known.

## 9. DNA Damage Modulates the Alternative Splicing of Genes Involved in the DDR

Genes involved in DNA repair, cell-cycle control and apoptosis use alternative splicing to expand their functional diversity. In the DNA repair category, splice variants of BRCA1 sensitize cells to DNA damage and alter repair mechanisms [213]. *Ercc1* produces a splice variant that increases sensitivity to cisplatin [214]. In cell-cycle genes, splice variants of CHK2 and the CDC25B phosphatase [215,216] display dominant-negative effects, while variants of cyclin D1 differentially regulate the DDR [10]. At least a dozen genes involved in apoptosis produce splice variants with distinct and sometimes opposite activities, including *Fas*, *Bcl-x*, *Mcl1*, *casp8* and *casp9* [217]. *Mdm2* produces splice variants that differentially control the activity of p53 and, hence, have a major impact on cell fate [218]. Coordination between repair, cell-cycle control and apoptosis is important to insure homeostasis and an efficient response to genotoxic stresses; thus we may anticipate common regulatory principles as well as cross-talks between molecular processes that are controlling splicing in these three functional categories. Apoptosis and cell-cycle control are already linked at the transcriptional level, for example, through the activity p53 and FOXO [219,220]. Signal transduction also serves to link cell-cycle control with the alternative splicing of apoptotic genes. For example, the AURKA kinase, which controls mitosis, converges on SRSF1 to prevent the production of pro-apoptotic variants of *Bcl-x*, *Mcl1* and *Casp9* [221]. Although this aspect has not yet been explored in detail, coordination between these functional categories likely includes the contribution of splicing factors and regulators. As mentioned above, EWS controls the alternative splicing of *cyclin D1*, of the apoptotic genes *Fas* and *Mdm2*, and of the DNA repair genes *Abl1* and *Chk2*. SRSF1 and Sam68 both control the alternative splicing of *Bcl-x* and *cyclin D1* [152,222,223,224]. HnRNP C controls the alternative splicing of *Bcl2l12*, *Bard1* and *Wrn* [130,131], while hnRNP A1 modulates the splicing of *Lrdd* and *Bard1* [131]. More complex regulatory strategies can arise since many splicing regulators lead alternate lives in the cytoplasm. For example, PTBP1 controls the splicing of *Fas*, but also promotes the translation of the CDK inhibitor p27Kip [34,225]. Based on these examples and the fact that several splicing regulators are modified or relocalized in response to DNA damage, many of the functional adjustments implemented to deal with DNA damage may occur through control of splicing.

Studies that have focused on a few genes or that have interrogated entire transcriptomes indicate that DNA damage has a broad impact on alternative splicing (Table 1 and Table 2). While the functional impact of these shifts is lacking in most cases, many of the genes affected are involved in DNA repair, cell-cycle control and apoptosis. In the DNA damage repair category, DNA lesions shift alternative splicing in *Atrip* [103], and improve the production of a splice variant encoding NBS1, a protein that interacts with the break-sensing MRE11/RAD50 complex [226]. In cell-cycle control genes, UV irradiation promotes splicing shifts in *Chek2*, *Mapk2* and *Abl1* [78]. UV and camptothecin also alter the alternative splicing of several genes involved in sensing/repair (e.g., *Atm*, *Atr*, *Chek1*, *Chek2*, *Parp2*, *Ddb1*, *Mlh1*, *Msh6*) and cell-cycle control (e.g., *Ccnb2*, *Aurka*, *Aurkb*, *Ccnt2*) [79,177] (Table 2).

**Table 1:** Alternative splicing events validated by RT-PCR that are affected by DNA damaging agents. The reference for each study is provided as a PubMed Identification (PMID) number.

Treatment	Cell Line	Affected Gene	PMID Number
5-Aza dC	MCF-7	*FN1*, *SYNE2*	25313066
Aclarubicin	SMA fibroblasts	*SMN2*	11734549
Amsacrine	U-937	*CASP2*	14757846
Arsenic (III) chloride	BEAS-2B	*GADD45*	18942077
Arsenite	AGS	*CD44*, *SFRS10 (TRA2B)*	19439532
Bleomycin (BLM)	HLE, HLF	*FIR*	24811221
BN80927 (TOP1, TOP2 inhibitor)	U-937	*CASP2*	14757846
Cadmium dichloride	RKO, EB-1	*PA26*	9926927
Camptothecin	HCT116	*AASDHPPT*, *APTX*, *BAT1*, *CASP2*, *CCT2*, *DDX17*, *EIF2S2*, *PNN*, *PPFIA1*, *PSMD12*, *RBM8A*, *RIOK1*, *RTN4*, *SF1*, *TCP1*, *WHSC1*, *ZRANB2*	20817775
	HeLa, U-937	*CASP2*	14757846, 18166155
	MCF-7	*CDC25C*, *FN1*, *SYNE2*, *HRAS*, *CHD2*, *EED*, *KIAA0232*, *MDM2*, *PAPOLG*, *RC3H2*, *THUMPD2*, *ZCCHC8*, *VEGF-A*, *RBM8A*, *SF1*	22871320, 25313066, 17709397, 20972445, 18086921, 20817775
	A431	*FOS* (generic splicing)	16921380
	Jurkat T lymphoma	*HNRPDL*, *IVNS1ABP*, *SF3B3*, *RUNX1*, *HMGXb4*, *SNRPB*, *SRSF2*	21163941
	HaCat, MDA-MB-231	*VEGF-A*	18086921
Capecitabine	MCF-7, HeLa S3, PA-1	*BCL2L1 (Bcl-x)*	18566212
Carboplatin	RTC	*BCL2L1 (Bcl-x)*	21198546
Chlorambucil	EcR293	*BCL2L1 (Bcl-x)*	18566212
Cisplatin	SH-SY5Y	*APAF1*, *H-RAS*	23613995
	Hep3B	*BCL2L1 (Bcl-x)*	19450518
	EcR293, MCF-7, HeLa S3, PC3, PA-1, SKOV-3	*BCL2L1 (Bcl-x)*	18566212
	HT1080	*BCL2L1 (Bcl-x)*, *PIG3*, *Smac/DIABLO*, *MDM2*	21327085, 25884497
	H358	*CASP8*	21157427
	MCF-7	*CDC25C*, *MDM4*, *MAGOH*, *AMZ2*, *CSDE1*, *EIF4A2*, *MDM2*, *MTA1*, *NFE2L1*, *STRAP*, *TMPO*, *VEGF*, *MDM2*	22871320, 18711402, 25884497, 25845590, 17018606
	AT5BIVA, MO59J	*HNRNPDL*	25884497
	HeLa S3, BT549, HDF1, MDA-MB-231, MG-63, MSU, RD, U2OS	*MDM2*	25845590, 25884497, 17018606
	H1299	*MDM2*, *MDM4*	17018606, 18711402
	Ishikawa	*MDM2*, *VEGF*	25884497
	HCT116, IMR90	*MDM4*	18711402
Cyclohexamide	U937	*CASP2*, *FAS*	15746654, 16131458
Cyclophosphamide	SKOV3	*BCL2L1 (Bcl-x)*	18566212
	H358	*BCL2L1 (Bcl-x)*, *CASP9*	18806759
Cytarabine	PC3	*BCL2L1 (Bcl-x)*	18566212
Dacarbazine	EcR293	*BCL2L1 (Bcl-x)*	18566212
Dactinomycin	EcR293, MCF-7, HeLa S3, PC3	*BCL2L1 (Bcl-x)*	18566212
	NIH3T3	*MDM2*	18469520
Daunorubicin	MCF-7, PC3, PA1	*BCL2L1 (Bcl-x)*	18566212
Diflomotecan	U-937	*CASP2*	14757846
Docetacel	HeLa S3	*BCL2L1 (Bcl-x)*	18566212
Doxorubicin	EU-3	*BIRC5*	15334064
	U-937, HCT116	*CASP2*	14757846, 20817775
	MDA-MB-231	*CDC25C*	22871320
	MCF-7	*CDC25C*, *PPM1D*	2287132, 18845566
	NIH3T3	*MDM2*	18469520
	EB-1, RKO	*PA26*	9926927
	T47D	*PPM1D*	18845566
Epirubicin	EcR293, MCF-7, HeLa S3, PC3	*BCL2L1 (Bcl-x)*	18566212
	HeLa, HL-60	*CASP2*	14757846, 12169392
	U937	*CASP2*, *FAS*	15746654, 16131458, 12169392, 14757846
	MCF-7	*CDC25C*	22871320
	U2OS	*NOXA*, *GADD45* (generic splicing)	21460037
Fluorouracil (5FU)	EcR293	*BCL2L1 (Bcl-x)*	18566212
Gemcitabine	A549	*BCL2L1 (Bcl-x)*, *CASP9*	11801602
	EcR293, MCF-7, PA-1, SKOV3, MiaPaCa2, PT45P1	*BCL2L1 (Bcl-x)*, *MKNK2*	18566212, 22797067
H2O2	Saos2	*ATF-3*	12034827
	HCT116	*SRSF3*	24284797
IDC92 (Indole derivative)	MDA-MB-435S	*RON*	20864806
Indolocarbazole derivative (NB-506)	P388	*BCL2L1 (Bcl-x)*, *CD44*, *SC35*, *STY*	11559564
Ionizing radiation (IR)	MCF-7	*BCL2L1 (Bcl-x)*, *CLU*	16465415, 15530543
	SH-SY5Y	*APAF1*, *H-RAS*	23613995
	LCL lymphoblastoid	*ASPM*, *FBXW7*, *GADD45G*, *MDM2*, *VWCE*	22039421
Ionizing radiation (IR)	Primary fibroblasts	*ATF-2*	12833146
	PBMCs	*NBS1*	18582154
	NF AG1519	*RAD17*	11602352
Irinotecan	U-937	*CASP2*	14757846
L-mimosin	MCF-7	*VEGF-A*	18086921
Methotrexate	EcR293, MCF-7, HeLa S3	*BCL2L1 (Bcl-x)*	18566212
Mitomycin C	PC3, U2OS, HTC116	*CD44*, *KSR1*, *IL24*	20110258, 17699766
	U2OS	*FAS*	18571879
	MCF-7, OVCAR3, SKOV3	*VEGF-A*	18086921, 25990504
Mitoxantrone	U-937	*CASP2*	14757846
Oxaliplatin	EcR293, MCF-7, HeLa S3, PC3, PA-1, SKOV-3	*BCL2L1 (Bcl-x)*	18566212, 20980256
	MCF-7	*MDM2*	25884497
Paclitaxel	U937	*CASP2*, *FAS*	15746654, 16131458
Paraquat	SH-SY5Y	*APAF1*, *BIN1*, *CASP9*, *CHN1*, *ERRC1*, *GNAO1*, *H-RAS*, *LMO3*, *NRG1*, *SKP2*, *SMN1*, *RPRD1A*	23613995, 21120952
Sodium arsenite	HeLa	*ABCG2*, *MGP*, *NCAM2*	25879800
	HCT116	*SFRS10*, *SRSF3*	24865968, 24284797
TAS-103 (TOP1, TOP2 inhibitor)	U-937	*CASP2*	14757846
Topotecan	EcR293, PC3	*BCL2L1 (Bcl-x)*	18566212
UV irradiation	HeLa	*ABL1*, *CHEK2*, *MAP4K2*, *MDM2*, *PIG3*	21816343, 25845590, 18801469
	HT1080	*BCL2L1 (Bcl-x)*, *PIG3*, *Smac/DIABLO*	21327085
	Human skin	*ELN*	19054052
	H1299	*MDM2*, *MDM4*	17018606
	MCF-7	*MDM2*, *MDM4*, *VEGF-A*, *PIG3*	25845590, 17018606, 18086921, 18801469
	MRC-5V1	*SRSF1*	21984412
UV-B irradiation	HaCat	*VEGF-A*	18086921
	MDA-MB-231	*VEGF-A*	18086921
UV-C irradiation	HeLa	*ADAR2*, *DDO*	15728250
	Hep3B	*BCL2L1 (Bcl-x)*, *CASP9*	19450518

Many splicing shifts caused by DNA damage occur in apoptotic genes. Cisplatin favors the production of pro-apoptotic splice variants of *c-flip*, *casp8*, *casp9* and *Bcl-x* [227]. While cisplatin and oxaliplatin modify *Bcl-x* splicing in different cells lines (Table 2), not all DNA damaging drugs (e.g., topotecan, etoposide, 5FU) have a similar impact on *Bcl-x* splicing, and these effects vary in different cell lines [151], Likewise, DNA damaging drugs alter the alternative splicing of other apoptotic genes but the sets of targets in different cell lines display little overlap [151] (Table 2). Cisplatin and oxaliplatin shift *Bcl-x* splicing in 293 cells in an ATM/CHK2-dependent manner [74]. UV irradiation promotes an ATM-independent shift in *Bcl-x* and *Mdm2* splicing in Hep3B cells and MCF-7 cells, respectively [79,228]. UV irradiation of human dermal fibroblasts fosters ATM-dependent changes in alternative splicing of genes that include *Atrip*, *Dnmt3a* and *Sirt3* [103]. Hundreds of alternative splicing changes occur when MCF-7 cells are treated with cisplatin [229], but these changes rely on the activation of the PI3K P110β and not the typical ATM and ATR signaling pathways. Overall, these results reinforce the notion that different treatments and different doses activate distinct pathways. Since the operational status of these pathways likely differs between cell lines, these variations will also be reflected in the identity of genes whose splicing and alternative splicing are affected by the treatments.

**Table 2:** Alternative splicing events affected by DNA damaging agents and identified by high-throughput screening. A web link address is provided with the source file. The reference for each study is provided as a PubMed Identification (PMID) number.

Treatment	Cell Line	Reference for Web Link	Source	PMID Number
Twenty chemotherapeutic drugs	EcR293, MCF-7, HeLa, PC3, PA-1, SKOV-3	[230]	Supplementary Information	18566212
Camptothecin	HCT116	[231]	Table S3	20817775
Camptothecin	Jurkat T lymphoma	[232]	Table S2	21163941
Camptothecin	MCF-7	[233]	Table S1	20972445
Cisplatin	MCF-7	[234]	File S4	25884497
Ionizing radiation (IR)	Lymphoblastoid cell lines, Primary fibroblasts	[235]	Table S8, Table S11	22039421
Sodium arsenite	HeLa	[236]	File S12, Table 6	25879800
UV-C irradiation	Hep3B	[237]	Table S2	19450518
UV-irradiation	Human dermal fibroblasts	[238]	Table S2	26106861
UV-B irradiation	Several	[239]	Table 2	18086921

ROS production also affects the alternative splicing of genes involved in the DDR (e.g., *Ercc1* for DNA repair, *Hras* and *Skp2* for cell-cycle control, and *Apaf1* and *Bin1* for apoptosis) [156]. Finally, bleomycin reduces the expression of SF3B1 and increases the production of a splice variant of FIR (aka PUF60, a U2AF2-related protein) that is deficient in its ability to confer transcriptional repression of *c-myc* [240]. The FIR proteins form a complex with SF3B1, which controls the splicing of *Fir* itself [240,241], of the cell-cycle gene *p27kip1* [240] and of *Bcl-x* [242]. The bleomycin-mediated drop in the expression of SF3B1 may therefore coordinate alternative splicing decisions to affect cell-cycle and apoptosis.

To determine if the impact of DNA damage on alternative splicing control preferentially affects the production of DDR components, we carried out an analysis using the PANTHER classification system [243]. We included in the analysis the splicing changes of six studies that used camptothecin, UV irradiation and sodium arsenate in different cell lines (Table 2). Notably, DNA damage elicited alternative splicing changes in genes that are preferentially associated with DNA repair, cell-cycle control and apoptotic signaling (Table 3), suggesting a concerted reprogramming of alternative splicing. Interestingly, an overrepresentation of splicing changes occurred in genes involved in splicing control, as previously noted by the group of Blencowe [180], and in genes involved in chromatin organization and modification. This last observation is interesting but not totally surprising given that chromatin coordinates DNA repair activities, and is now known to be intimately associated with the control of splice site selection.

**Table 3:** DNA damage-induced changes in alternative splicing occur preferentially in genes implicated in DNA repair, cell-cycle control and apoptosis. We compiled alternative splicing changes occurring in 2214 genes from six studies that used camptothecin, UV and sodium arsenate (PMID number 20817775, 21163941, 20972445, 25879800, 19450518 and 26106861; see Table 3). The annotation to biological processes was carried out using the PANTHER bioinformatics platform [243]. Relative to the distribution of 20,814 human genes in each process, PANTHER identified processes that were enriched in genes whose splicing is affected by DNA damage (the number of genes affected is indicated). The statistical significance of the enrichment, expressed as a *p* value (Bonferroni corrected), is indicated for each process. *p* Values inferior to 0.01 were observed for 3.4% (268/7812) of processes. Of these, 36 processes displayed a *p* value of less than 0.001 and a gene enrichment greater than 2-fold. Nineteen processes from this set are listed below.

GO Biological Process Complete	Number of Genes	Fold Enrichment	*p* Value
DNA repair	94	2.29	4.04E−09
cell cycle checkpoint	58	2.4	2.09E−05
cell cycle phase transition	64	2.18	1.19E−04
mitotic cell cycle phase transition	63	2.18	1.70E−04
mitotic cell cycle phase	57	2.05	5.09E−04
mitotic cell cycle process	138	2.04	1.00E−10
cell cycle phase	57	2.04	6.26E−04
mitotic cell cycle	51	2.03	6.87E−12
intrinsic apoptotic signaling pathway	41	2.49	1.72E−04
apoptotic signaling pathway	75	2.25	1.85E−06
RNA splicing	93	2.92	3.69E−15
RNA splicing, via transesterification reactions	68	3.19	3.00E−12
mRNA splicing, via spliceosome	67	3.19	5.26E−12
chromatin modification	117	2.42	1.29E−13
covalent chromatin modification	75	2.55	6.99E−09
chromatin organization	123	2.1	4.28E−10
chromosome organization	167	2.04	1.31E−13
histone modification	74	2.54	1.18E−08
regulation of gene expression, epigenetic	49	2.39	4.28E−04

## 10. Conclusions

Recent efforts have helped establish the multiple ways through which DNA damage alters the activity of splicing factors and modulates constitutive and alternative splicing. DNA lesions attract splicing factors that participate in mounting the first steps of the DDR. Occasionally, some of these factors (e.g., BRCA1, BCLAF1, PSF and hnRNP C) play an even more direct role in damage repair. Other splicing proteins are recruited at sites of DNA damage, only to be tagged with post-translational modifications that affect their localization and/or activity. Yet other splicing factors are modified by enzymes that dissociate from sites of damage, such as the CHK1 and CHK2 kinases. These alterations affect splicing in different ways. For example, DDR-specific splicing complexes can be formed to improve splicing, or specific interactions can be disrupted to prevent the normal coupling of transcription with splicing. The splicing changes that often occur in genes involved in damage sensing, DNA repair, cell-cycle control and apoptosis therefore has the potential to feedback on every step of the DDR. However, despite the increasing number of examples documenting the global impact of DNA damage on splicing and alternative splicing, the functional impact of most of these changes remains to be evaluated. The variety of DNA damaging agents, their doses and the identity of cell lines selected to carry out studies also limit the usefulness of comparing the splicing response in different systems. Different types of damage require the contribution of distinct repair machineries, whose operational status may vary between cell lines. The baseline expression of DDR components may also vary considerably between genomically unstable cancer cell lines (often lacking p53) and immortalized ones. The complexity created by the various combinations of treatment and cell lines will hopefully be mitigated by the continuous development of high-throughput procedures and bioinformatics tools to help distill the common and specific rules that guide the interconnections between DNA damage and the splicing response.

Striving to obtain a complete description of the splicing-relevant alterations elicited by DNA damage has the potential to improve anti-cancer strategies. Anti-cancer treatments often aim to overwhelm the DNA repair machinery as a means of triggering apoptosis. While the intrinsically high genomic instability of cancer cells makes them more dependent on enhanced DNA repair activities, hence contributing to anti-cancer drug resistance, this reliance also provides a therapeutic opportunity because inactivating one or several DDR components may sensitize cancer cells to DNA damaging agents [244,245]. This potential was first illustrated by BRCA1, a DDR factor that also directly contributes to splicing [182]. Some BRCA1 mutations increase sensitivity to DNA damaging agents [246], and the inactivation of both BRCA1 and PARP1 is lethal [247]. Many splicing factors implicated in the DDR are also mutated or aberrantly expressed in cancer. For example, genes that encode U2AF, SF3B1 and SRSF2 are mutated in myelodysplastic syndromes [248,249], while others promote cancer or provide a cancer-specific metabolic signature [250,251]. It is revealing that the depletion of RBMX, EWS, FUS, SKIP and Tra2 all augment DNA damage-induced apoptosis [77,108,123,185], and that knocking down PTBP1 abolishes resistance to genotoxic drugs in pancreatic cancer cells [252]. Thus, small molecules that target generic spliceosomal components [253] or specific splicing regulators [254] may be offering new strategies to combat cancer and chemoresistance.

The stochastic accumulation of DNA damage caused by replication errors, intrinsic metabolic and external mutagens has also been associated with organismal and stem cell aging [255]. The persistence of unrepaired DNA lesions and the resulting chronic DDR activation cause a permanent cell-cycle arrest that defines cellular senescence [256,257]. Aging and senescence are associated with changes in chromatin structure, in the expression of splicing factors and in alternative splicing [257,258,259,260]. The diversity of factors that affect the splicing of DDR genes and the activity of the splice variant itself are providing a large selection of novel targets that can potentially be used to prevent stem cell senescence and impair cancer development in aging individuals.
